# Mechanisms of D1/D2-like dopaminergic agonist, rotigotine, on lower urinary tract function in rat model of Parkinson’s disease

**DOI:** 10.1038/s41598-022-08612-3

**Published:** 2022-03-16

**Authors:** Mifuka Ouchi, Takeya Kitta, Hiroki Chiba, Madoka Higuchi, Mio Togo, Yui Abe-Takahashi, Nobuo Shinohara

**Affiliations:** Department of Renal and Genitourinary Surgery, Graduate School of Medical Science, Hokkaido University, Kita 15 Nishi 7, Kita-ku, Sapporo, Hokkaido 060-8638 Japan

**Keywords:** Parkinson's disease, Urology

## Abstract

Parkinson’s disease (PD) is a neurodegenerative condition caused by the loss of dopaminergic neurons in the substantia nigra pars compacta. As activation of dopaminergic receptors is fundamentally involved in the micturition reflex in PD, the objective of this study was to determine the effect of a single dose of rotigotine ([−]2-(N-propyl-N-2-thienylethylamino)-5-hydroxytetralin) on intercontraction interval (ICI) and voiding pressure (VP) in a rat model of PD. We used 27 female rats, PD was induced by injecting 6-hydroxydopamine (6-OHDA; 8 μg in 2 μL of 0.9% saline containing 0.3% ascorbic acid), and rotigotine was administrated at doses of 0.125, 0.25, or 0.5 mg/kg, either intravenous or subcutaneous injection. In rats with 6-OHDA-induced PD, intravenous injection of 0.25 or 0.5 mg/kg rotigotine led to a significantly lower ICI than after vehicle injection (*p* < 0.05). Additionally, VP was significantly lower in animals administered rotigotine compared to those injected with vehicle (*p* < 0.05). Compared to vehicle-injected animals, subcutaneous administration of rotigotine (0.125, 0.25, or 0.5 mg/kg) led to a significantly higher ICI at 2 h after injection (*p* < 0.05); however, there was no change in ICI after injection with (+)-SCH23390 hydrochloride. Dermal administration of rotigotine in a rat model of PD could suppress an overactive bladder.

## Introduction

Parkinson’s disease (PD) is a chronic neurodegenerative condition caused by the loss of dopaminergic neurons in the substantia nigra pars compacta. The number of PD patients who are older than 50 years is expected to double by 2030, compared to 2005, due to an aging population and greater life expectancy^1^. PD is characterized by both motor and non-motor symptoms, and a recent review has demonstrated that the prevalence of lower urinary tract symptoms (LUTS) ranges from 27 to 63.9% in patients with PD^2^. Among non-motor symptoms, such as constipation, REM sleep behavior disorder, hyposmia, fatigue, and urinary symptoms, autonomic neuropathy has been reported to greatly impair patients’ quality of life. Specifically, an overactive bladder is seen even in the early stages of PD^3^ and one study has reported that istradefylline, an adenosine A2A receptor antagonist, can effectively improve not only motor symptoms but also LUTS in patients with PD in clinical settings^4^.

Normal micturition cycles, which consist of filling and emptying, are maintained through coordination between the bladder and the urethra. Cystometry has demonstrated that the detrusor muscle, supplied by the inferior hypogastric plexus, maintains low pressure within the bladder, and that the urethral sphincter, supplied by the inferior hypogastric plexus and the pudendal nerve, closes the bladder outlet during the filling phase. This coordinated activity in the lower urinary tract is mediated by the central nervous system, including the brain and the spinal cord, and the supraspinal site, which projects into the pontine micturition center (PMC), plays an important role in controlling bladder activity^5^.

A previous study has reported that the micturition reflex is accelerated in a 6-hydroxydopamine (6-OHDA)-induced PD model^6,7^ and that various anti-parkinsonian drugs affect the micturition reflex in this model; specifically, while D1 and D5 receptor agonists can antagonize the micturition reflex (bladder hyperactivity), D2 and D4 agonists have a role in stimulating the reflex^8^.

Rotigotine ([-]2-(N-propyl-N-2-thienylethylamino)-5-hydroxytetraline) is a dopamine agonist with an affinity for all dopamine receptors (D1 to D5), which when delivered daily as a single dose through a transdermal patch, provides consistent and stable plasma concentrations over the next 24 h^9,10^. The effects of rotigotine have been confirmed in clinical settings, which translate to an improvement in detrusor-related overactive bladder in patients with PD^11^. Therefore, we investigated the direct effect of rotigotine on the micturition reflex in a rat model of PD.

## Results

ICI and VP values after administration of vehicle or rotigotine (0.125, 0.25, or 0.5 mg/kg i.v.) are shown in Table 1A,B, respectively.

**Table 1:** Intravenous administration of rotigotine in Parkinson’s disease model.

Dose of injection (mg/kg)	Inter contraction interval	*p* value
**(A)**		
Vehicle (n = 3)	12 min 11 s ± 2 min 40 s	
0.125 (n = 3)	3 min 40 s ± 0 min 36 s	0.08
0.25 (n = 3)	1 min 35 s ± 0 min 8 s*	0.018
0.5 (n = 3)	1 min 29 s ± 0 min 16 s*	0.029

Dose of injection (mg/kg)	Voiding pressure	*p* value
**(B)**		
Vehicle (n = 3)	39.61 ± 2.95	
0.125 (n = 3)	27.57 ± 2.32	0.115
0.25 (n = 3)	25.69 ± 1.07	0.069
0.5 (n = 3)	22.26 ± 3.21*	0.028

(A) Intercontraction interval (n = 3), (B) Voiding pressure (cmH_2_O) (n = 3).

*Significant difference versus vehicle (*p* < 0.05). Data are shown as mean ± SE.

In 6-OHDA-treated rats, intravenous injection of 0.25 or 0.5 mg/kg rotigotine led to a significantly lower ICI than after vehicle injection (*p* < 0.05); however, 0.125 mg/kg i.v rotigotine did not significantly affect ICI. Intravenous administration of rotigotine led to significantly lower VP values than after vehicle injection (*p* < 0.05).

Subcutaneous injection of rotigotine (0.125, 0.25, or 0.5 mg/kg) and (+)-SCH23390 hydrochloride (1.0 mg/kg), a D1/D5 receptor antagonist, in PD rats led to a significantly higher ICI at 2 h than after vehicle injection [0.125 mg/kg: 9 min 58 s ± 1 min 51 s vs. 12 min 7 s ± 2 min 0 s (*p* = 0.004); 0.25 mg/kg: 9 min 41 s ± 1 min 20 s vs. 10 min 15 s ± 2 min 35 s (*p* = 0.042); 0.5 mg/kg: 9 min 30 s ± 2 min 3 s vs. 12 min 15 s ± 2 min 19 s (*p* = 0.002)] (Table 2A).

**Table 2:** Subcutaneous administration of rotigotine in Parkinson’s disease model.

Dose of injection	Vehicle	1 h after rotigotine injection	2 h after rotigotine injection
**(A)**			
0.125 mg/kg (n = 7)	9 min 58 s ± 1 min 51 s	13 min 7 s ± 1 min 51 s*	12 min 7 s ± 2 min 00 s*
0.25 mg/kg (n = 8)	9 min 41 s ± 1 min 20 s	8 min 22 s ± 1 min 38 s	10 min 15 s ± 2 min 35 s*^†^
0.5 mg/kg (n = 9)	9 min 30 s ± 2 min 3 s	10 min 18 s ± 2 min 4 s	12 min 15 s ± 2 min 19 s*^†^

Dose of injection	Vehicle	1 h after rotigotine injection	2 h after rotigotine injection
**(B)**			
0.125 mg/kg (n = 7)	23.1 ± 2.0	24.2 ± 1.3	22.5 ± 1.1
0.25 mg/kg (n = 8)	38.8 ± 1.7	38.9 ± 2.0	38.5 ± 2.3
0.5 mg/kg (n = 9)	36.7 ± 2.8	33.8 ± 2.4	30.4 ± 2.2

Dose of injection	Vehicle	2 h after rotigotine injection	D1 antagonist injection
**(C)**			
0.25 mg/kg (n = 8)	9 min 54 s ± 1 min 17 s	13 min 05 s ± 1 min 32 s*	15 min 27 s ± 2 min 8 s

(A) Inter-contraction interval (minutes), (B) Voiding pressure (cmH_2_O), (C) Subcutaneous administration of rotigotine and D1 antagonist injection in Parkinson’s disease model.

*Significant difference versus vehicle (*p* < 0.05).

^†^Significant difference versus 1 h after rotigotine injection. Data are shown as mean ± SE.

ICI was 10 min 11 s ± 4 min 22 s before vehicle injection, 9 min 16 s ± 3 min 15 s at 1 h and 10 min 6 s ± 4 min 28 s at 2 h after vehicle injection. VP values did not change at 1 and 2 h after subcutaneous injection of 0.125, 0.25, or 0.5 mg/kg rotigotine (Table 2B). There were no significant differences between ICI values at 2 h after rotigotine injection and (+)-SCH23390 hydrochloride (Table 2C) and VP remained unaffected. Residual urine volume did not change significantly after intravenous or subcutaneous injections. post-void residual volume (PRV) ranged from 0.01 to 0.25 mL.

## Discussion

This is the first report showing that subcutaneously administered rotigotine can inhibit bladder activity in a rat model of PD by potentially activating D1/D5 (D1-like) dopamine receptors, and that ICI varies based on route of rotigotine administration, i.e., it significantly decreased following intravenous administration but increased following subcutaneous injection. These contrasting effects of rotigotine may be attributed to complete agonism of the dopamine receptor family (D1, D2, D3, D4, and D5) in the micturition reflex and the affinity of dopamine receptors.

Previous studies in 6-OHDA-treated rats have demonstrated detrusor hyperactivity^8^, indicating that degeneration of dopaminergic neurons in the substantia nigra pars compacta can cause an overactive bladder. In fact, compared to healthy animals, bladder contraction was elicited at a significantly smaller volume threshold in a primate model of PD induced by l-methyl-4-phenyl-l,2,3,6-tetrahydropyridine^12^. Further, 6-OHDA-treated rats exhibited significantly smaller bladder capacity during micturition, as measured by cystometry^8^, which is consistent with our data on ICI in PD rats.

The pharmacokinetic characteristics of rotigotine include a median T_max_ at 4–8 h following a single subcutaneous injection of 3, 6, or 12 mg/kg in the plasma of rats. But, given the opposing changes in micturition reflex upon two different routes of rotigotine administration described here, we speculate that, following subcutaneous administration, rotigotine may stimulate more D1-like dopamine receptors as if it were a D1/D5 dopamine receptor agonist. In contrast, when administered intravenously, rotigotine may have enhanced affinity for D2/D3/D4 (D2-like) dopamine receptors, mimicking a D2/D3/D4 dopamine receptor agonist. This divergence can be accounted for by the high affinity of rotigotine for D1, D2, and D3 receptors (pEC_50_ of 9.6 ± 0.1, 10.4 ± 0.1 and 8.2 ± 0.2, respectively) and a relatively lower affinity for D4 and D5 receptors (pEC_50_, 7.7 ± 0.1 and 7.7 ± 0.2, respectively)^13^. An additional mechanism may involve D2-like dopamine receptors in a high affinity state^14^, which would be affected by low concentrations of dopamine.

Previous reports of dopamine agonists on micturition in 6-OHDA-treated rats show that, compared to ICI of 6.2 ± 0.4 min in controls, a dose of 0.5 mg/kg significantly increased ICI to 7.9 ± 0.5 min and to 9.3 ± 0.7 min at 1.0 mg/kg; in contrast, a D2-like receptor agonist significantly reduced ICI to 4.1 ± 0.5 min at 0.2 mg/kg and 2.3 ± 0.3 min at 0.4 mg/kg compared to 6.3 ± 0.6 min in control animals^8^. These previous findings suggest robust D2-like receptor activation immediately after injection and gradual D1-like receptor activation, probably due to differences in the level of affinity, blood concentration, and/or the effect of the dopamine family on the micturition reflex. Similarly, another report has stated that a D2-like agonist seemed to reduce bladder capacity due to worsening of detrusor overactivity in patients with PD^15^. Therefore, rotigotine may predominantly act as a D1-like dopamine receptor agonist rather than as a D2-like receptor agonist, thereby affecting the micturition reflex. Moreover, apomorphine, an anti-parkinsonian drug, has a higher affinity for dopamine D2 receptors and displays a dose- and time-dependent biphasic effect on bladder filling^16^. Additionally, low-dose apomorphine induced an initial increase in micturition volume, while medium- and high-dose apomorphine first decreased micturition volume and subsequently increased it. As an improvement in clinical motility and bladder function due to rotigotine has been reported in PD^11,17^, we speculated that transdermal delivery of rotigotine can improve bladder function in clinical use.

We demonstrate significantly greater ICI values after subcutaneous administration of rotigotine compared to vehicle injection in PD rats; however, no change was seen after injection with (+)-SCH23390 hydrochloride, which is a D1 antagonist. Thus, we hypothesized that ICI increases after subcutaneous rotigotine injection but that it decreases after (+)-SCH23390 hydrochloride, and hence, administered the antagonist only after confirming changes in ICI at 2 h after subcutaneous rotigotine injection.

Dopamine released from the substantia nigra and the striatum can inhibit bladder contraction by stimulating D1 receptors on striatal neurons related to bladder storage; however, dopamine depletion due to degeneration of the neurons of the substantia nigra and the striatum can reduce such stimulation of D1 receptors, resulting in impaired bladder storage. In PD, bladder hyperactivity is suppressed by D1 agonists, and compared to sham rats, bladder activity was facilitated in a PD model, indicating that dopamine depletion can cause bladder hyperactivity^4,6,8^. It has been reported that D1 agonists can inhibit the micturition reflex in a rat model of PD, suggesting that D1-like dopamine receptors may inhibit the micturition reflex^8^. (+)-SCH23390 hydrochloride facilitates the micturition reflex in normal conscious rats^18^, and congruently, inhibition of bladder hyperactivity was reversed by intracerebroventricular administration of SCH23390 hydrochloride after the micturition reflex was inhibited by stimulation of the substantia nigra pars compacta in cats^19^. Thus, the increase in ICI following rotigotine administration may be due to its effect on D1-like dopamine receptors. In contrast to a previous report where (+)-SCH23390 hydrochloride facilitated micturition reflex^18^, no changes were observed upon the use of a D1-like dopamine receptor antagonist in our experiment even though both dose and time were optimized based on pharmacokinetics^18,20^. SKF 38,393, a D1 agonist, has been shown to be effective in inhibiting bladder hyperactivity^8^ and other neurotransmitters might be involved in regulating bladder function in PD. Notably, clinical and animal studies have not been able to completely elucidate lower urinary tract dysfunction in PD, and the results of the current study can be attributed to the complexity of dopamine receptor activation.

It is known that stimulation of dopamine D2 and D4 receptors, but not the D3 receptor, can facilitate the micturition reflex, whereas stimulation of D1 and D5 receptors can inhibit the micturition reflex^8^. Rotigotine is a unique dopamine agonist with an affinity for all dopamine receptor subtypes, which may be because while other neurotransmitters bind to the site projecting into the PMC, including γ-aminobutyric acid (GABA) and/or adenosine. Further, an in vivo microdialysis study by Kitta et al. demonstrated that a GABAergic mechanism is involved in the midbrain periaqueductal gray projection to the PMC^21^. The micturition reflex is under dopaminergic regulation through D1 receptors with potential GABAergic involvement. Micturition-induced inhibition of GABA was not observed in rat model of PD, and 6-OHDA-treated rats exhibit bladder hyperactivity, which mimics facilitation of the micturition reflex induced by D1 receptor blockade.

In the -supraspinal pathway under normal conditions, tonic activation of dopaminergic neurons in the substantia nigra pars compacta activates D1-like dopamine receptors expressed on GABAergic inhibitory neurons in the striatum to inhibit the micturition reflex. In contrast, dopaminergic neurons in the substantia nigra pars compacta are lost in PD, leading to the loss of D1-like dopamine receptor activation and reduced activation of inhibitory GABAergic neurons in the striatum^22^. A prolonged micturition reflex after subcutaneous rotigotine administration in this study prompted us to hypothesize that rotigotine-induced inhibition of micturition reflux was due, at least in part, to D1-like dopamine receptor agonism. Hence, we examined the potential involvement of D1-like dopamine receptors in rotigotine-induced inhibition of micturition reflux. To prove this hypothesis, we deem it important to investigate the effects of GABA in future studies. Similarly, adenosine A2A receptor antagonism significantly increases ICI compared to pre-administration in PD rats, indicating that the adenosine A2A receptor may be involved in bladder overactivity^6^. Also, it has been reported that rotigotine may have higher binding affinity for 5-HT receptors^23,24^.

A clinical study investigating the effect of rotigotine on bladder function in PD patients reported that bladder overactivity was suppressed, probably due to stimulation of D1 receptors^11^. Our basic findings show that subcutaneous administration of rotigotine significantly improved urinary symptoms in a rat model of PD, which supports results from previous clinical studies. The rotigotine transdermal patch (Neupro®) is routinely used in the clinic after being approved by the European Medicines Agency in 2006 and the Food and Drug Administration in 2007. It is well-known that PD is characterized by a profound disruption of motor function, including bradykinesia, tremor, rigidity, perturbed gait, and postural instability^3^. A meta-analysis found that rotigotine, launched as a dopamine receptor agonist, can reduce motor dysfunction and improve activities of daily living when used in the treatment of idiopathic PD^25^. As PD symptoms are caused by a massive loss of dopaminergic neurons from the substantia nigra pars compacta, rotigotine would improve not only motor symptoms but also micturition-related symptoms after transdermal administration, apart from its overall effects to ameliorate pollakiuria in PD patients.

## Conclusion

We provide corroborating evidence that an overactive bladder is suppressed by the administration of rotigotine in rat model of PD and that transdermal administration, rather than an intravenous route, is critical for the control of the micturition reflex. Importantly, as this mechanism could have a role in activating D1-like dopamine receptors, transdermal administration of rotigotine could be effective in improving not only motor symptoms but also an overactive bladder in patients with PD.

## Methods

### Animals

Female Sprague–Dawley rats (n = 27), aged 12–14 weeks and weighing 250–375 g, were used in this study. All experiments conformed to National Institutes of Health animal care guidelines. This study was approved by the Hokkaido university ethics committee (approval number 18-0098) and conformed to ARRIVE guidelines.

### PD model rats

Rats were intraperitoneally injected with 60 mg/kg of pentobarbital (Kyoritsuseiyaku Corporation, Tokyo, Japan) before surgery for producing unilateral 6-OHDA-induced lesions. 6-OHDA (8 μg in 2 μL of 0.9% saline containing 0.3% ascorbic acid) was microinjected unilaterally into the left substantia nigra pars compacta of the brain at a point 5.3 mm posterior to the bregma, 8.0 mm ventral to the dura, and 2.2 mm laterally^6^. Additionally, 6-OHDA (10 μg) was injected into the lateral striatum of rats before intravesical pressure was measured to obtain a rat model of unilateral PD. Motor dysfunction was confirmed in these animals to establish PD.

### Surgical procedures

Rats were anesthetized with isoflurane (Pfizer Japan Inc., Tokyo, Japan) and urethane (1.1 g/kg subcutaneous injection, Wako Pure Chemical Industries, Ltd., Osaka, Japan). The lower abdomen was dissected, polyethylene tubing (Becton Dickinson™, Franklin Lakes, USA) was inserted into the bladder dome to record bladder filling and pressure, and then ligated using thread. After confirming that no liquid was leaking from the bladder dome, the abdominal wound was sutured. Rats were allowed to stabilize for approximately 2 h before the experimental protocol was initiated.

### Measurement of intercontraction interval (ICI) and voiding pressure (VP)

**Figure 1:**
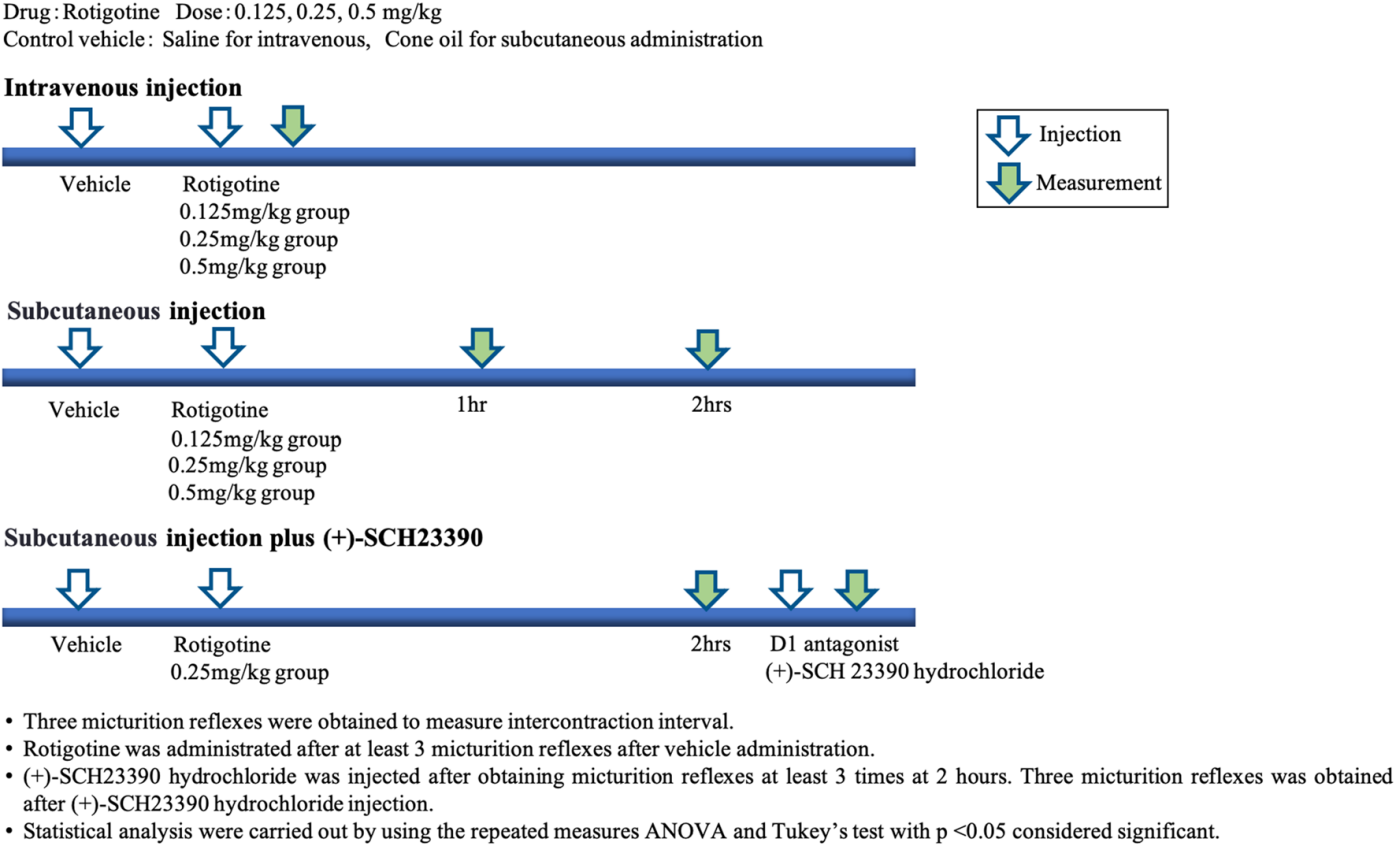
Data collection following intravenous and subcutaneous administration in Parkinson’s disease rats.

ICI and VP for micturition were measured using cystometry (Fig. 1), as previously reported^26^.

ICI was defined as time-to-contraction under constant filling conditions and VP was defined as maximum intravesical pressure obtained upon detrusor contractility for micturition. In addition, PRV was measured by withdrawing intravesical fluid through the catheter using gravity before drug injection and at the end of the micturition reflex after drug administration. PRV was measured to confirm that changes in ICI caused by the drugs were not due to increased PRV. The bladder was emptied using a syringe after micturition to measure residual volume. For cystometry, a catheter from the bladder was connected via a three-way stopcock to a pressure transducer for monitoring bladder pressure. Saline at room temperature was continuously infused into the bladder using a syringe pump (TE-332S, Terumo, Japan) at a constant rate (3 mL/h). These parameters were monitored by a pressure amplifier, incorporated into a personal computer via a PowerLab system analog-to-digital converter (AD Instruments, Nagoya, Japan), and recorded on chart data acquisition software at a sampling rate of 400 Hz. Cystometry to assess the effects of drug administration was initiated after stable bladder pressure baseline data were obtained for 60 min.

### Drug administration

**Figure 2:**
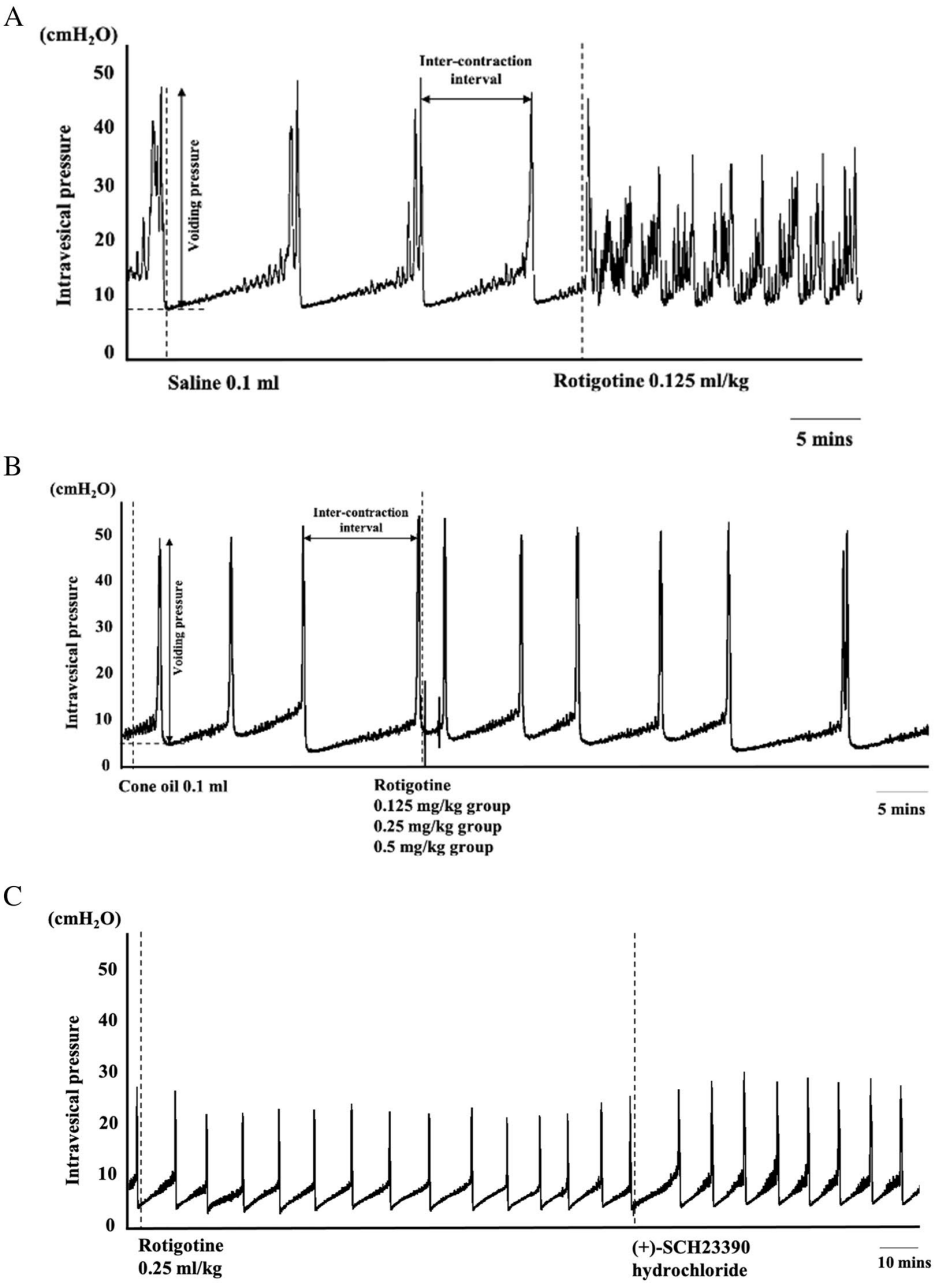
(**A**) Intravenous administration of rotigotine. (**B**) Subcutaneous administration of rotigotine. (**C**) Cystometory trace for subcutaneous rotigotine treatment with or without SCH23390.

Rotigotine (Sigma–Aldrich, St. Louis, MO) was administrated cumulatively, either intravenously or subcutaneously, to a rat model of PD. Cystometry was conducted 1–2 h after inserting the bladder catheter and was based on previous experiments of bladder function in parkinsonian rats^8^. Intravenous rotigotine was administered at least 3 micturition reflexes after vehicle injection. Drugs for intravenous administration were dissolved in saline (0.9% w/v sodium chloride; Otsuka Pharmaceutical Factory, Inc., Tokushima, Japan) (Fig. 2A).

Cystometry was performed after at least three micturition reflexes post (+)-SCH23390 injection, which was dissolved in cone oil (Wako Pure Chemical Industries, Ltd.) for subcutaneous administration (Fig. 2B).

Rotigotine doses used were 0.125, 0.25, and 0.5 mg/kg for both intravenous and subcutaneous administration. (+)-SCH23390 hydrochloride (Sigma–Aldrich, St. Louis, MO) was intravenously administrated after at least 3 micturition reflexes and at 2 h after rotigotine injection (0.25 mg/kg s.c.) (Fig. 2C).

Saline (0.1 mL/kg i.v.) was administered as the intravenous vehicle while cone oil (0.1 mL/kg s.c) was administered as a subcutaneous vehicle. (+)-SCH23390 hydrochloride was administered intravenously at 1.0 mg/kg. All doses were determined based on previous reports^18^.

### Statistical analysis

The results of urinary variables, such as ICI and VP, are expressed as the mean ± standard error, and data in the PD model were statistically analyzed using repeated analysis of variance and Tukey’s test; *p* < 0.05 was considered significant.
